# Heterogeneity in Response during Multisystemic Therapy: Exploring Subgroups and Predictors

**DOI:** 10.1007/s10802-016-0242-9

**Published:** 2016-12-29

**Authors:** Esther C. A. Mertens, Maja Deković, Jessica J. Asscher, Willeke A. Manders

**Affiliations:** 1 Department of Child and Adolescent Studies, Utrecht University, P.O. Box 80140, 3508 TC Utrecht, The Netherlands; 2 Research Centre for Forensic Child and Youth Care Sciences, University of Amsterdam, Nieuwe Achtergracht 127, 1018 WS Amsterdam, The Netherlands; 3 Youth Spot, Amsterdam University of Applied Sciences, Wibautstraat 5a, 1091 GH Amsterdam, The Netherlands

**Keywords:** MST, Trajectories, Parental sense of competence, Prosocial peers, Externalizing problems

## Abstract

Multiple studies have shown that Multisystemic Therapy (MST) is, at group level, an effective treatment for adolescents showing serious externalizing problem behavior. The current study expands previous research on MST by, first, examining whether subgroups of participants who respond differently to treatment could be identified. Second, we investigated if the different trajectories of change during MST could be predicted by individual (hostile attributions) and contextual (parental sense of parenting competence and deviant and prosocial peer involvement) pre-treatment factors. Participants were 147 adolescents (mean age = 15.91 years, 104 (71%) boys) and their parents who received MST. Pre-treatment assessment of the predictors and 5 monthly assessments of externalizing behavior during treatment took place using both adolescent and parents’ self-reports. Six distinct subgroups, showing different trajectories of change in externalizing problem behavior during MST, were identified. Two of the 6 trajectories of change showed a poor treatment response, as one class did not change in externalizing problem behavior and the other class even increased. The remaining 4 trajectories displayed a positive effect of MST, by showing a decrease in externalizing behavior. Most of these trajectories could be predicted by parental sense of parenting competence. Additionally, lower involvement with prosocial peers was a predictor of the group that appeared to be resistant to MST. Adolescents do respond differently to MST, which indicates the importance of personalizing treatment. Protective factors, such as parental sense of parenting competence and prosocial peers, seem to require additional attention in the first phase of MST.

Many treatments have been developed to decrease externalizing problem behavior, as this behavior has been found to negatively affect individuals, peers, families and communities. One of these treatments is Multisystemic Therapy (MST). MST is based on Bronfenbrenner’s social ecological model which states that behavior is determined by interaction between multiple systems (i.e., family, school, peers and neighborhood) in which the adolescent is nested (Bronfenbrenner 1979). In accordance with this model, MST aims to address the multi-determined nature of externalizing problem behavior at individual, peer, family, school and community levels. Since it is important to change behavior in the context in which it occurs, MST uses a home- and community-based treatment. Thus, therapeutic sessions are implemented in the environment in which the problem behavior occurs (e.g., in homes or schools; e.g., Curtis et al. 2009).

Multiple studies in different countries have shown that MST is an effective treatment (see for overview Henggeler 2011). In most of these studies, the effectiveness of MST was examined in a traditional way, that is by comparing the average change in externalizing behavior of the MST group with the average change in the control group. In this approach, the treatment effects are considered homogeneous for the total group of participants and the treatment is reduced to a binary condition in which it is effective or not. However, the impact of a treatment can differ per individual. By solely analyzing the average, important variations across individuals within a group are sacrificed (Na et al. 2015).

Evidence supporting the notion that there are subgroups who differ in their response to treatment comes from studies that examined moderators of treatment effectiveness (e.g., Manders et al. 2013). These studies have shown that pre-treatment differences between participants in both demographic (such as age, gender, ethnicity) and more substantive characteristics (such as initial level of problems, participants’ personality, and quality of external support) do affect the impact of the treatment, with participants who share certain characteristics benefitting more than others from the same treatment (Kaminski et al. 2008). The studies that examined such moderator effects provided valuable insights into differential responsiveness to treatment, however in these studies the subgroup membership is defined by shared pre-treatment characteristics, rather than by similarities on the outcome of interest (Lennon et al. 2005). To better specify the impact of a treatment, a person-oriented approach is necessary to identify heterogeneity within groups (i.e., subgroups who show different trajectories of change in the outcome). This approach focuses on relations among individuals instead of between variables as in a variable-oriented approach (Muthén and Muthén 2000). In the present study we used such a person-oriented approach, namely Latent Growth Mixture Modeling (LGMM), to chart heterogeneity in trajectories of change in adolescents’ externalizing problems during MST.

To our knowledge, only one study examined trajectories of change in adolescents who, following psychiatric crisis, received either MST or psychiatric hospitalization (Halliday-Boykins et al. 2004). In this study, five trajectories, based on change in externalizing and internalizing symptoms over 16 months following crisis, were identified. In three of those trajectories the symptoms were stable over time and the groups differed only in the initial levels of symptoms: High unimproved, borderline unimproved and subclinical group. The remaining two trajectories showed decreases in symptoms over time: High improved and borderline-improved group. The findings suggest that a substantial proportion of adolescents sustained high levels of symptoms. This study, however, focused on adolescents who experienced high levels of psychiatric symptoms that warrant psychiatric hospitalization, rather than on adolescents who show externalizing problems (traditionally, the target group of MST). Moreover, the trajectories were based on the whole sample, without differentiating between the group that received MST and the control group that received psychiatric hospitalization. It is, therefore, not possible to determine the pattern of improvement in the MST group only.

In the present study, using the data from an Randomized Controlled Trial (RCT) on effectiveness of MST in The Netherlands (e.g., Asscher et al. 2013), we specifically focus on adolescents who received MST due to serious externalizing problems and we examine if there were different latent classes regarding their response to the treatment. Given the findings of Halliday-Boykins et al. (2004) and findings of several other recent studies that examined variation in treatment effects across individuals (e.g., Fowler et al. 2014; Kellam et al. 2014), we expected to find heterogeneity among adolescents in their response to MST, including a subgroup that responds poorly to treatment since all examples above found such a resistant group. However, given the scarcity of studies that examined this question, no specific number of subgroups was hypothesized.

A second aim of this study was to examine pre-treatment predictors of the different trajectories of change in externalizing problem behavior. Examining factors that predict different responses to treatment has important clinical implications, as factors that predict non-improvement or even deterioration during treatment deserve special attention at the beginning of the treatment. Criteria for selecting the predictors were as follows: First, they had to fit in the theoretical framework of MST and it had to be reasonable to expect that they might affect treatment outcome. Second, they had to be identified as predictors of child and adolescent treatment outcomes in previous meta-analyses and treatment studies (e.g., Crean and Johnson 2013; De Haan et al. 2013; Reyno and McGrath 2006). Third, the factors had to be dynamic (i.e., factors that can be changed during the treatment) rather than static factors that cannot be changed during treatment (e.g., gender, IQ, ethnicity). This will make the results clinically relevant since the therapist can actually address identified factors that predict less beneficial trajectories early during MST in order to improve the trajectory of change of that specific adolescent. Based on these criteria, predictors from three domains were selected: An adolescent characteristic (i.e., hostile attribution bias), parenting (i.e., parental sense of competence), and peer relations (i.e., involvement with deviant and prosocial peers). Due to statistical power, only a limited number of predictors could be analyzed.

Hostile attribution bias, an individual characteristic, indicates the tendency to attribute hostile intentions to others in ambiguous situations more often than other adolescents do. Due to this hostile attribution, the adolescent is more likely to respond aggressively compared to when the adolescent perceives the intentions of the other as benign or accidental (Orobio de Castro et al. 2002). This more aggressive way of responding has been proposed as predictor of a more negative treatment outcome (Boxer et al. 2015). Adolescents who have hostile attribution biases are less capable of understanding and discussing their own and others’ emotions, which negatively influences the development of communicative skills, inhibition of behavior and self-control. This results in an increase in aggressive and violent solutions; as these adolescents are not able to think of alternative reactions, evaluate the responses and select the preferred option (Crean and Johnson 2013). Crean and Johnson (2013) have found that a decrease in hostile attribution bias is a predictor for reducing externalizing behavior in the intervention Promoting Alternative Thinking Strategies which aims to enhance emotional and social competencies in order to decrease externalizing behavior. Additionally, Hudley and Graham (1993) showed that a decrease in hostile attribution bias is a predictor of decrease in aggressive behavior during a cognitive intervention aimed to change cognitions in order to diminish aggressive behavior. Therefore, it may be that the participants in the subgroup that shows a small decrease in externalizing behavior score high on hostile attributions.

Regarding the parenting domain, parental sense of competence concerning their parenting may have an influence on the response of the participants to the treatment. Sense of parental competence concerns the belief of parents in their own capability to effectively manage parenting tasks (De Haan et al. 2009). Parents with a high sense of parental competence are warmer, more accepting and encourage autonomy of their children (Gondoli and Silverberg 1997). They show effective parenting which decreases the adolescent’s externalizing behavior (Deković et al. 2012; Jones and Prinz 2005). In contrast, parents with a low sense of parental competence feel inadequate and helpless, show less effective parenting, withdraw from interactions with the adolescent and give up addressing behavioral problems (Coleman and Karraker 1997). Their difficulties in acquiring new strategies to improve their parenting has a negative effect on MST outcome (Huey et al. 2000). The meta-analysis of De Haan et al. (2013) showed that low levels of parental sense of competence was a predictor for negative treatment outcomes. Parents with high levels of parental sense of competence might choose to put a lot of effort into the treatment and might be more persistent in applying the knowledge learned during MST. Previous research on MST has shown that a higher parental sense of competence appears to be an important predictor of positive changes in parenting which, in turn, decreases the adolescent’s externalizing behavior (Deković et al. 2012). Therefore, it is expected that the participants in the subgroup that shows a large decrease in externalizing behavior have parents with a high sense of parental competence.

Concerning the domain of peer relations, the response to MST could differ based on the involvement with deviant or prosocial peers. When an adolescent is involved with deviant peers, externalizing problem behavior is reinforced by these peers resulting in a maladaptive socialization process (Deater-Deckard 2001). Less deviant peer involvement has been found to be a predictor of positive outcome in MST (Tiernan et al. 2015). Likewise, Boxer (2011) found that involvement with deviant peers decreased the probability that the adolescent would finish MST successfully. Frequent contact with deviant peers was a predictor for treatment drop-out (De Haan et al. 2013). Thus, the subgroup which shows a small decrease in externalizing behavior may be more involved with deviant peers. Prosocial peers oppose and refrain from externalizing problem behavior (Osgood et al. 2013). Therefore, affiliation with prosocial peers might work as a buffer against the development of externalizing behavior (Deater-Deckard 2001). This is supported by Huey et al. (2000) who stated that more engagement with prosocial peers and less affiliation with deviant peers might be important mechanisms during MST to reduce delinquent behavior. In the classroom-based Good Behavior Game intervention –which aims to promote prosocial behavior and diminish antisocial behavior– more affiliation with prosocial peers was a predictor of decreases in antisocial behavior (Van Lier et al. 2005). Thus, the subgroup showing a large decrease in externalizing behavior may affiliate more with prosocial peers.

In sum, the present study expands previous work by exploring heterogeneity in treatment response to MST on an individual level and examining predictors of the adolescents’ trajectories of change. Researching the trajectories of change during treatment improves the understanding of “what works for whom” and may help adjusting treatments to the needs of specific subgroups. Additionally, identifying individual trajectories may be useful for therapists working with juveniles by helping them to understand the different processes of change that take place during treatment. By identifying child, parental and contextual attributes that might influence treatment outcome, therapists can monitor or address these factors early during the treatment to improve the trajectory of change concerning externalizing behavior. Hence, the results of this study might show which pre-treatment factors deserve additional attention in MST so that the treatment can be tailored to the different subgroups.

## Method

### Participants and Procedure

In total, the sample consisted of 147 adolescents and their parents who participated in an RCT (Dutch Trial register number: 1390) on the effectiveness of MST (Asscher et al. 2013). The adolescents were referred by referring agencies (i.e., Child Protection Council, juvenile judges, Bureaus Youth Care, local referral institutions) to MST due to severe, persistent and violent antisocial behavior. When MST was considered to be suitable for the family, the referrers informed the juveniles and their families that a study was being conducted to research the effectiveness of youth care. If the families met the inclusion criteria for MST according to the MST supervisors of the participating institutions, research procedures were explained to the juveniles and their families and their informed consent to participate in the study was obtained by researchers. The institutional review board and the Medical Ethic Committee of Utrecht University approved the design of the study.

Data were collected by trained research assistants. The assessment before the start of the treatment took place in the homes of the participants. Additionally, adolescent externalizing behavior was measured monthly during MST through a telephone interview with adolescents and parents separately. These telephone interviews lasted about 15 to 20 min. Each family member received 10 Euros for completing the pre-treatment assessment.

Adolescents’ ages at the start of MST ranged between 12 and 18 with an average age of 15.91 (*SD* = 1.42). Of the adolescents 104 (71%) were boys. Furthermore, 74 (51%) adolescents had a Dutch ethnicity. Adolescents belonging to an ethnic minority mostly had a Moroccan ethnicity (20%) or a Surinamese ethnicity (16%). All adolescents from an ethnic minority backgrounds spoke fluently Dutch. However, not all parents spoke Dutch. In those cases a translator was present during MST. The questionnaires were translated for this group by research assistants native in Berber or in Turkish.

### MST

MST addresses several ecological systems in which the adolescent is embedded: Family, school, peers and neighborhood. The treatment lasts typically 4 to 6 months and is tailored to the needs of specific clients. MST is provided in the homes of the participants, but sessions can also be given in schools, neighborhood settings or social service agencies. Treatment goals are formulated in consultation with the family. Tasks are assigned to accomplish these goals and progress is monitored regularly in family sessions (Asscher et al. 2013; Deković et al. 2012).

In this study, MST was terminated on average after 5.72 months (*SD* = 1.90). Six teams with in total 30 therapists of three MST institutions provided MST. Of these therapists, 59% was men, 10% had an ethnic minority background, 68% had a master’s degree and 41% followed additional training in cognitive behavioral therapy and/or family system therapy (Deković et al. 2012). The therapists had a low caseload with on average 4.65 families per therapist.

Treatment integrity was assessed with the Therapist Adherence Measure (TAM) consisting of 15 items. It measures the adherence of the therapist to the nine principles of MST. Via a telephone interview parents rated the items monthly on a five-point Likert-type scale (*1 = Not at all* to *5 = Very much*). Therapist adherence as experienced by a family during treatment was indicated by the average score per family. The mean adherence score was satisfactory (*M* = 4.36, *SD* = 0.51) and comparable to adherence scores found in American studies (Asscher et al. 2013; Deković et al. 2012).

### Measurements

#### Externalizing Behavior

Parents and adolescents were asked monthly during MST whether or not the adolescent had shown certain behavior described in each of the items (1 *= true,* 2 *= false*) during the last month. For the parents, this assessment existed of four items of the Externalizing problems scale from the Child Behavior Checklist (Achenbach 1991). Adolescents were asked five items from the adolescent version, the Youth Self Report. The items that were selected tap each construct most adequately based on face validity and examination of items’ factor loadings in previous studies (Asscher et al. 2013; Deković et al. 2012). The composite score for externalizing behavior was computed as the mean of both scores. Convergent validity of this shortened version was good with a correlation of 0.55 (*p* < 0.001) between the first monthly measurement and the premeasurement of externalizing behavior (i.e., the total CBCL combined with the total YSR) and 0.58 (*p* < 0.001) between the last monthly measurement and the post measurement of externalizing behavior (i.e., the total CBCL combined with the total YSR). The internal consistency (Cronbach’s alphas) across five assessments ranged from 0.76 to 0.83.

#### Hostile Attributions

The adolescents’ hostile attributions were measured at the start of MST with the Hostility subscale of the Children’s Automatic Thought Questionnaire filled in by the adolescent (Schniering and Rapee 2004). This subscale consisted of 10 items (e.g., “When someone hurts me, I have the right to hurt that person”) on which they indicated how many times they had that specific thought the past week on a five-point Likert-type scale (0 *= not at all* to 5 *= all the time*). This scale showed good psychometric properties (Schniering and Rapee 2004). Cronbach’s alpha was 0.74.

#### Parental Sense of Competence

Parents’ sense of competence concerning parenting was assessed using a scale from the Parenting Stress Index (Abidin 1983) which is one of the most often used instruments to assess this concept (Jones and Prinz 2005). Parents filled this questionnaire in at the start of MST. This scale consisted of 15 items (e.g., “Had the feeling that I cannot cope with parenting” – reversed coded) answered on a six-point Likert scale (1 *= I totally disagree* to 6 *= I totally agree*). It has strong psychometric properties (Abidin 1983; Haskett et al. 2006). Cronbach’s alpha was 0.86.

#### Involvement with Deviant Peers

The amount of deviance in the adolescent’s peer network was assessed with the Trouble subscale (four items) of the Family, Friends, and Self Scale (Simpson and McBride 1992) and with the Deviant peers subscale (seven items) of the Basic Peer Questionnaire (Weerman and Smeenk 2005) filled in by the adolescent at the start of MST. The Family, Friends, and Self Scale is highly reliable and validated multiple times (Henggeler et al. 1999). The Basic Peer Questionnaire was found to measure peer affiliation equally well as indirect measures asking participant’s peers (Weerman and Smeenk 2005). All 11 items were answered on a five-point Likert-type scale (1 *= none* to 5 *= almost all*). The internal consistency was 0.91.

#### Involvement with Prosocial Peers

Adolescent’s amount of prosocial behavior in the adolescent’s peer network was measured with seven items of the Family, Friends, and Self Scale (Simpson and McBride 1992) filled in by the adolescent at the start of MST. The items were answered on a five-point Likert-type scale (1 *= none* to 5 *= almost all*). The internal consistency was 0.84.

### Analytic Strategy

Missing data were examined for the whole RCT sample (i.e., including the control group that was not included in this study; Asscher et al. 2013). This indicated that participants with missing data on the post measurement did not significantly differ on any assessed variable from those retained. The Little’s test for missing completely at random showed that data were missing completely at random, χ^2^ (3097) = 3200.556, *p* = 0.095. On the monthly measurements of adolescent and of parent reported externalizing behavior there were no significant differences between participants with and without missing data concerning adolescents’ gender, adolescents’ ethnicity and pre- and post-measurements of adolescents’ and parental reported externalizing behavior except for two monthly measurements. On the third monthly measurement the group with missing parental reported data scored higher on self-reports and parental reports of externalizing behavior at premeasurement, than the group without missing parental data. These differences were small, *F* self-reported (1, 145) = 7.84, *p* = 0.006, η^2^_partial_ = 0.051, *F* parental reported (1, 145) = 8.59, *p* = 0.004, η^2^_partial_ = 0.056. On the fourth monthly measurement the group with missing self-reported data contained more Moroccan and Surinamese adolescents than the group without missing data. This difference was small, χ^2^ (7) = 19.99, *p* = 0.006, φ = 0.018. The Little’s test for missing completely at random indicated that data on the monthly measurements were missing completely at random, χ^2^ (254) = 270.42, *p* = 0.229. Therefore, missing data were handled with multiple imputations carried out by the expected maximization algorithm conducted in LISREL 8.8 (Deković et al. 2012; Graham 2009).

In order to determine whether non-independence of the families treated by the same therapist might be a concern, we calculated the design effect, following Muthén (2000), as *d* = 1 + ρ (*c* – 1), where ρ is the average ICC (0.03) and *c* is the common cluster size (i.e., the average number of families per therapist, 4.65). The design effect was 1.11. This is considered small enough to ignore since a design effect smaller than 2.0 is considered acceptable (Muthén and Satorra 1995). Thus, non-independence of data was no concern and no multilevel analyses were necessary.

To explore heterogeneous trajectories concerning participants’ responses to MST, a Latent Growth Mixture Model (LGMM) was conducted using M*plus* version 7.2. The participants are probabilistically assigned to subpopulations of latent classes based on the data of the growth model. LGMM allows variances and co-variances in intercepts and slopes between latent classes as well as within latent classes. These variances were modeled as it is reasonable to assume that not all individuals within one latent class have exactly the same intercept and slope. So, participants within a latent class could deviate from the estimated mean growth trajectory of that specific latent class (Jung and Wickrama 2008).

First, the change in externalizing behavior was established using Latent Growth Curve (LGC) modeling. Then, Latent Class Growth Analyses (LCGA) were conducted with increasing numbers of classes for orientation on the data. LCGA is a more restricted model of LGMM, as no variance within latent classes is allowed, only between classes. This makes the model less complex. Therefore, it is helpful for the analyses to begin with LCGA and proceed with LGMM (Jung and Wickrama 2008).^1^ Next, LGMM models were tested with an ascending number of classes. The maximum likelihood estimation with robust standard errors (MLR) was used to correct for non-normality in the variables. Iterations and random starts were increased to make sure that the found solution was not caused by a local maximum. The final model was chosen based on a low Baysian Information Criteria (BIC), a high entropy, a significant improvement in model fit based on the Bootstrapped Likelihood Ratio Test (BLRT), theoretical justification and the usefulness and interpretability of the trajectories (Connell and Frye 2006; Nagin and Odgers 2010).

To analyze which factors were predictors of the latent classes found, the four predictors were added to the selected model using the three-step approach. In the first step the LGMM model is estimated without taking the predictors into consideration. Second, a new variable is created representing the probability of belonging to each of the classes for each participant. Hence, this variable also takes the misclassification into account. Third, this new variable is regressed on the predictor variables. With this method the predictors have no influence on the determination of the latent classes and are only used to predict class membership of participants. The three-step approach works well if the entropy of the model has a value above 0.6 (Asparouhov and Muthén 2014).

## Results

### Change in Externalizing Behavior

To examine how externalizing behavior changed during treatment, LCG analyses were conducted. The linear growth model showed a good fit, BIC = −1007.57, CFI = 0.984, RMSEA = 0.076. In this model, externalizing behavior showed a linear decrease during MST, confirming that adolescents as a group improve during MST.

### Determination of the Number of Latent Classes

To explore how many different trajectories of response to MST could be identified, LGMM models were conducted. For the LGMM models with three classes or more the variance of the slope was fixed in all classes due to the negative definite of the slope in the covariance matrices. Fixing of the slope is not unusual as LGMM is a complex model (e.g., Galatzer-Levy et al. 2013). The model fit statistics of the models are shown in Table 1. The BIC value did not change much when classes were added, but the entropy improved and the BLRT was significant. In the seven-classes solution the moderate-decreasers class was split into two almost parallel classes that were substantively non-distinct. Therefore, this model was not chosen even though the BLRT indicated that the seven-classes solution fitted the data significantly better than the six-classes solution. In the six-classes solution a new class appeared which increased in externalizing behavior. This class is theoretically interesting. The two-classes solution seemed to fit the data equally well as the six-classes solution based on the BIC and entropy. However, the second class contained only seven participants with relatively differing trajectories of change concerning their externalizing problem behavior. Hence, the group was not theoretical justifiable, nor interpretable, and the BLRT showed that more classes fitted the data significantly better. So, based on a high entropy, a significant BLRT and the theoretical usefulness of the increasing class, the LGMM model with six classes was chosen as final model.

**Table 1:** The fit information for the seven models (*N* = 147)

	LGMM				
	AIC	Adjusted BIC	BIC	Entrop	BLRT
M1	-1037.474	-1039.216	-1007.570	-	-
M2	-1058.838	-1061.101	-1019.962	0.921	< 0.001
M3^a^	-1065.110	-1067.547	-1023.244	0.734	< 0.001
M4^a^	-1069.341	-1072.301	-1018.504	0.747	0.040
M5^a^	-1078.516	-1081.998	-1018.708	0.839	< 0.001
M6^a^	-1086.633	-1090.638	-1017.853	0.860	< 0.001
M7^a^	-1096.785	-1101.311	-1019.033	0.917	0.013*

*LGMM* Latent growth mixture model, *M* model, *AIC* Akaike information criterion, *BIC* Bayesian information criterion, *BLRT* Bootstrap likelihood ratio test

^a^The variance of the slope was fixed for all classes in the model

*Two out of 80 bootstrap draws did not converge

**Fig. 1:**
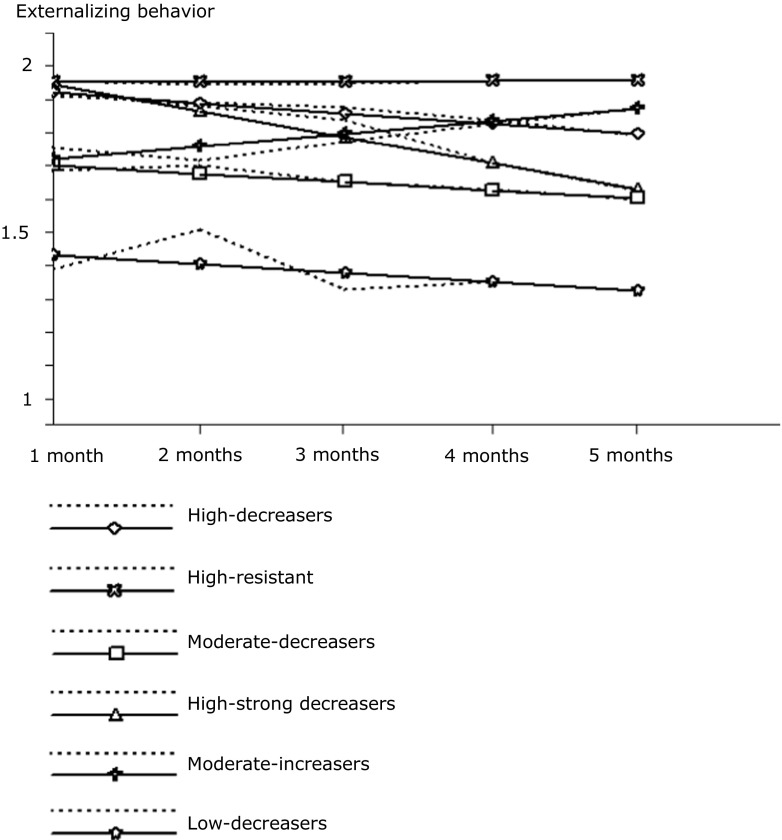
Observed and estimated trajectories of the six latent classes in the final LGMM model in which dotted lines represent the observed trajectories and solid lines the estimated trajectories

The largest class, high-decreasers (37.4%), demonstrated an initial high score on externalizing behavior which decreased during the treatment. The second largest class, high- resistant (28.6%), was characterized by an initial high score on externalizing behavior which remained stable during MST. The third class, moderate-decreasers (16.3%), scored initially moderately on externalizing behavior which decreased during the treatment. The fourth class, high-strong decreasers (8.2%), showed an initial high score on externalizing behavior which decreased strongly during the treatment. The fifth class, moderate-increasers (6.1%), was characterized by an initial moderate score on externalizing behavior which increased in this behavior during the treatment. The smallest class, low-decreasers (3.4%), demonstrated an initial relatively low score on externalizing behavior which decreased during the treatment. The means and slopes of the six classes are presented in Table 2 and the observed and estimated trajectories are graphically displayed in Fig. 1. Overall, two of the six trajectories of change showed a poor treatment response, as one class did not change in externalizing problem behavior and the other class even increased. The other four trajectories displayed a positive effect of MST, since they all decreased in their externalizing behavior.

**Table 2:** Descriptives, demographics and predictors (*N* = 147) of the six latent classes

	High-decreasers	High-resistant	Moderate-decreasers	High-strong decreasers	Moderate-increasers	Low-decreasers
	(*n* = 55)	(*n* = 42)	(*n* = 24)	(*n* = 12)	(*n* = 9)	(*n* = 5)
Intercept (SE)	1.920 (0.015) ***	1.951 (0.014)***	1.702 (0.024)***	1.943 (0.035)***	1.721 (0.047)***	1.431 (0.062)***
Slope (SE)	-0.031 (0.003)***	0.001 (0.003)	-0.025 (0.006)***	-0.079 (0.006)***	0.037 (0.011)**	-0.026 (0.011)*
Age	15.87 (1.38)	15.81 (1.52)	16.26 (1.30)	15.87 (1.17)	15.82 (1.97)	15.62 (1.29)
Percent male	67.3%	73.8%	66.7%	75.0%	88.9%	60.0%
Percent Dutch	45.3%	46.3%	62.5%	66.7%	55.6%	60.0%
Hostile attributions	4.09 (0.73)	4.16 (0.69)	4.12 (0.71)	3.95 (0.47)	4.03 (0.66)	3.94 (0.84)
Sense of competence	1.87 (0.85)	1.62 (0.86)	2.32 (0.73)	2.57 (0.85)	2.39 (0.61)	2.39 (0.87)
Deviant peers	4.27 (0.68)	4.33 (0.63)	4.00 (0.69)	3.97 (1.05)	3.75 (1.34)	3.97 (0.50)
Prosocial peers	2.81 (0.90)	2.62 (0.57)	3.18 (0.86)	3.05 (0.85)	3.11 (1.18)	3.03 (0.99)

*p < 0.05 **p < 0.01. ***p < 0.001

### Predictors of the Latent Classes

The descriptives of the six latent classes are presented in Table 2. No significant differences were found between the latent classes on demographic variables, *F* age (5, 141) = 0.39, *p* = 0.853, η^2^_partial_ = 0.01, χ^2^gender (5) = 2.52, *p* = 0.774, φ = 0.13, χ^2^ethnicity (30) = 35.26, *p* = 0.233, φ = 0.50.

To analyze which predictors differentiated between the latent classes, a multinomial logistic regression analysis was conducted (Table 3). The parents of the high-strong decreasers, the moderate-decreasers and the moderate-increasers scored significantly higher on sense of competence than the parents of the high-resistant class. The parents of the high- strong decreasers reported significantly more sense of competence than parents of the high- decreasers. The moderate-decreasers had parents with significantly higher sense of parenting competence than parents of the high-decreasers. Furthermore, the moderate-decreasers had significantly more involvement with prosocial peers than the high-resistant class. There were no significant differences concerning involvement with prosocial peers among the other classes. Moreover, there were no significant differences between the classes regarding hostile attributions and involvement with deviant peers.

**Table 3:** Results multinomial logistic regression analysis (*N* = 147) for the six latent classes

	Hostile attributions		Sense of competence		Deviant peers		Prosocial peers	
	OR	B (SE)	OR	B (SE)	OR	B (SE)	OR	B (SE)
High-decreasers vs.								
High-resistant	1.07	0.06 (0.39)	0.72	-0.33 (0.31)	1.00	0.00 (0.41)	0.71	-0.34 (0.29)
Moderate-decreasers	1.30	0.26 (0.47)	2.16*	0.77 (0.37)	0.63	-0.46 (0.47)	1.78	0.58 (0.37)
High-strong decreasers	0.92	-0.08 (0.40)	3.20*	1.16 (0.49)	0.64	-0.45 (0.52)	1.49	0.40 (0.51)
Moderate-increasers	1.00	-0.01 (0.72)	2.58	0.95 (0.52)	0.37	-0.98 (0.74)	2.69	0.99 (1.46)
Low-decreasers	0.92	-0.08 (0.70)	2.23	0.80 (0.70)	0.61	-0.50 (0.64)	1.36	0.31 (0.80)
High-resistant vs								
Moderate-decreasers	1.22	0.20 (0.43)	2.98**	1.09 (0.37)	0.63	-0.46 (0.42)	2.50**	0.92 (0.35)
High-strong decreasers	0.87	-0.15 (0.39)	4.42**	1.49 (0.49)	0.64	-0.45 (0.48)	2.09	0.74 (0.50)
Moderate-increasers	0.94	-0.07 (0.69)	3.56*	1.27 (0.50)	0.37	-0.98 (0.71)	3.78	1.33 (1.50)
Low-decreasers	0.87	-0.14 (0.70)	3.08	1.13 (0.71)	0.61	-0.50 (0.64)	1.91	0.65 (0.81)
Moderate-decreasers vs.								
High-strong decreasers	0.71	-0.34 (0.44)	1.48	0.40 (0.51)	1.01	0.01 (0.46)	0.84	-0.18 (0.53)
Moderate-increasers	0.77	-0.27 (0.71)	1.20	0.18 (0.46)	0.59	-0.52 (0.54)	1.51	0.41 (1.39)
Low-decreasers	0.71	-0.34 (0.71)	1.03	0.03 (0.71)	0.96	-0.04 (0.53)	0.76	-0.27 (0.82)
High-strong decreasers vs.								
Moderate-increasers	1.08	0.08 (0.65)	0.81	-0.22 (0.61)	0.59	-0.53 (0.68)	1.81	0.59 (1.42)
Low-decreasers	1.00	0.00 (0.67)	0.70	-0.36 (0.78)	0.96	-0.05 (0.62)	0.91	-0.09 (0.87)
Moderate-increasers vs								
Low-decreasers	0.93	-0.08 (0.86)	0.87	-0.15 (0.77)	1.62	0.49 (0.71)	0.51	-0.68 (1.58)

*OR* Odds Ratio

*p < 0.05. **p < 0.01

## Discussion

The current study expanded previous research by examining whether subgroups of participants who respond differently to MST could be identified. The results showed that there were six subgroups. Although findings, at the group level, showed that MST is generally effective in decreasing externalizing problems, two of the six subgroups showed a poor treatment response. One, relatively large, subgroup maintained high levels of externalizing behavior throughout the treatment, and thus appeared to be resistant to MST (high-resistant), whereas the other, small, subgroup even showed an increase in externalizing behavior during MST (the moderate-increasers). The finding that a substantive percentage of the adolescents did not evidently decrease in problems corresponds with the results found by Halliday-Boykins et al. (2004). Two thirds of the adolescents did show a positive treatment response. The improvement did not seem to depend on the initial level of problems. One subgroup had high levels of externalizing behavior at the beginning and this behavior decreased strongly during MST (the high-strong decreasers). Three other subgroups showed a gradual decrease in externalizing behavior during treatment one of which initially showed high levels of externalizing behavior (the high-decreasers), another moderate levels of this behavior (the moderate-decreasers) and the third low levels (the low-decreasers).

Next, it was explored whether individual (hostile attributions) and contextual (parental sense of parenting competence, and involvement with deviant and prosocial peers) pre- treatment factors could predict the trajectories of change in externalizing problem behavior during the treatment. Hostile attributions in the beginning of MST did not predict the subgroups. A possible explanation for this nonsignificant finding might be that hostile attributions are particularly relevant for only a specific type of externalizing behavior. Hostile attribution bias correlates with reactive aggression, but not with proactive aggression. Proactive aggression is related to delinquency, whereas reactive aggression is associated with impulsivity and anger in response to threat (Bailey and Ostrov 2008; Walters 2007). Even though both types of aggression can be found among individuals with externalizing problem behavior, it is possible that proactive aggression was more prevalent than reactive aggression in this sample making hostile attribution bias less relevant.

In contrast to other studies that found effects of deviant peer involvement on the outcome of MST, the present study showed no differences between the subgroups regarding involvement with deviant peers. The main difference between the present study and those studies reporting significant effects was the assessment of deviant peer involvement. In the present study this involvement was self-reported by the adolescent with 11 items. Huey et al. (2000) assessed this predictor with three items reported by parents. Boxer (2011) measured this using referral description which probably represents the perception of the parents or the therapist. It might be that parents and therapists do not have a correct view of the adolescent’s friends. Hence, reports from these informants might be distorted especially when measured with only a few items. Tiernan et al. (2015) assessed deviant peer involvement in the last 30 days when the adolescent was on average 9.8 weeks in treatment. Consequently, deviant peer affiliation was measured concerning a period in which the adolescent already received MST. In our study, on the other hand, deviant peer involvement was assessed at the start of MST, so that the treatment had not yet influenced the adolescent. In sum, it might be that no effect of deviant peer affiliation was found due to the type and timing of measurement of deviant peer involvement that was used.

Involvement with prosocial peers appeared to work as a protective factor for some subgroups, as having many prosocial friends increased the chance of belonging to the moderate-decreasers instead of belonging to the high-resistant subgroup. This protective function of prosocial peers was also found in the study of Deater-Deckard (2001). The positive effect of prosocial peers is thus not a mirror effect of involvement with deviant peers, as no differences between the groups were found concerning deviant peers. It might be that the social competence of the adolescents with moderate levels of externalizing problem behavior is better developed than that of adolescents showing high levels of externalizing behavior, since higher levels of social competence is associated with lower levels of externalizing behavior. Adolescents with adequate social competence are more capable of building relationships with prosocial peers (Stepp et al. 2011). Hence, it is easier for prosocial peers to affiliate with these moderately deviant peers than with highly deviant peers. The affiliation with prosocial peers might lead to a stronger decrease in externalizing problem behavior. This finding, however, should be interpreted with caution, as the significant effect of prosocial peer involvement was not systematically found between subgroups.

Concerning parental sense of competence, the results were less clear-cut. In general, it seems that parental sense of competence serves as a protective factor. The parents of the high- resistant group showed the lowest level of parenting competence at the beginning of MST, and the higher the sense of parental competence, the more beneficial the trajectory of the adolescent was regarding their level of externalizing behavior. The results build on the findings of Deković et al. (2012) who found that MST increased parental sense of competence leading to positive changes in parenting which ultimately led to a decrease in adolescents’ externalizing problem behaviors. Hence, it appears that parental sense of competence can predict the adolescent’s response to MST as well as mediate the treatment effect. A high level of parental sense of competence therefore might lead to more involvement in treatment which has been related to better behavioral outcomes than when parents are not involved (e.g., Pereira et al. 2016).

The moderate increasers are an exception to this, since their parents showed relatively high levels of parental sense of competence even though it is a disadvantageous trajectory. Interestingly, Halliday-Boykins et al. (2004) reported a similar finding: Higher levels of caregiver empowerment increased the likelihood that the adolescent would belong to an unimproved group. It is possible that, in this group, parents overestimate their sense of competence and/or attribute their adolescents’ problems solely to the adolescents themselves, rather then, at least partly, to their own parental role. Those parents may be less open to recommendation of the therapist and less willing to get involved in treatment. Although clearly the role of parental sense of competence in predicting treatment response warrens further study, the present finding suggests that therapists should pay special attention to this at the beginning of the therapy and try to explore how parents feel about their own competence as parents.

When assessing the significance of these results, it is important to consider the strengths and weaknesses of the current study. A strength is that the study was conducted in a naturalistic setting. Additionally, there were monthly measures of the adolescents’ externalizing behaviors during MST which allowed the modeling of treatment response as therapy progresses. Furthermore, the externalizing behavior was assessed by multiple informants. A limitation of the present study was the sample size. Even though the sample size was relatively large for a treatment study (Weisz et al. 2005), it was still quite small for the complex LGMM models. Additionally, a limited number of predictors could be analyzed. Although the selected predictors seem most relevant for MST trajectories of change, there may be more predictors worth considering. Another consequence of the small sample size was that two subgroups were relatively small. This made the statistical comparison between the subgroups more difficult, as a difference must be very large in order to gain statistical significance. Given the overall effectiveness of MST (e.g., Henggeler 2011), it is to be expected that the group showing an increase, rather than a decrease in externalizing behavior (i.e., moderate-increasers) is relatively small. This is, however, a theoretically very interesting group that deserves attention in future research. Nevertheless, small subgroups are not rare in LGMM and the two subgroups contained more than 2% of the total sample which is considered to be acceptable (Galatzer-Levy et al. 2013). Finally, it should be noticed that in the present study we focused on pre-treatment factors, because of their clinical relevance, since factors that predict non-improvement or even deteriorations during treatment deserve special attention at the beginning of the treatment. Those factors, however, are also explicit targets of MST. It is possible that the *changes* in those factors from pre- to post-treatment are even better predictors of the treatment outcome than the initial levels at the treatment outset.

Notwithstanding these limitations, this study shows that not all adolescents respond the same way to treatment which seems to be predicted by their parents’ sense of competence and, partly, by involvement with prosocial peers. This underpins the importance of taking the social ecological environment into account which is the basis of MST. The present study is the first step in analyzing heterogeneity in response to MST and examining the differences between these subgroups. By examining pre-treatment factors that predict differences in response to treatment, adolescents at risk of non-improvement or even deterioration in behavior can be identified. Due to the changeable characteristics of the examined predictors, therapists might address and attempt to change the factors relevant for that specific individual early in treatment. Additional attention to key factors of the treatment can be given from the beginning so that the trajectories of change concerning externalizing behavior might improve and thus increase the effectiveness of MST for that individual. Therefore, at the beginning of MST extra attention should be paid to the protective factors when the adolescent is at risk of following a disadvantageous trajectory of change.

## Footnotes

1 The results of the LCGA are available on request from the first author.
